# BMD-Related Genetic Risk Scores Predict Site-Specific Fractures as Well as Trabecular and Cortical Bone Microstructure

**DOI:** 10.1210/clinem/dgaa082

**Published:** 2020-02-18

**Authors:** Maria Nethander, Ulrika Pettersson-Kymmer, Liesbeth Vandenput, Mattias Lorentzon, Magnus Karlsson, Dan Mellström, Claes Ohlsson

**Affiliations:** 1 Centre for Bone and Arthritis Research, Department of Internal Medicine and Clinical Nutrition, Institute of Medicine, Sahlgrenska Academy, University of Gothenburg, Gothenburg, Sweden; 2 Bioinformatics Core Facility, Sahlgrenska Academy, University of Gothenburg, Gothenburg, Sweden; 3 Clinical Pharmacology, Department of Pharmacology and Clinical Neuroscience, Umea University, Umea, Sweden; 4 Geriatric Medicine, Institute of Medicine, Sahlgrenska Academy, University of Gothenburg, Gothenburg, Sweden; 5 Mary MacKillop Institute for Health Research, Australian Catholic University, Melbourne, Australia; 6 Clinical and Molecular Osteoporosis Research Unit, Department of Orthopedics and Clinical Sciences, Lund University, Skåne University Hospital, Malmö, Sweden; 7 Sahlgrenska University Hospital, Department of Drug Treatment, Gothenburg, Sweden

**Keywords:** bone mineral density, genetic risk scores, fractures, bone microstructure, trabecular, cortical

## Abstract

**Context:**

It is important to identify patients at highest risk of fractures.

**Objective:**

To compare the separate and combined performances of bone-related genetic risk scores (GRSs) for prediction of forearm, hip and vertebral fractures separately, as well as of trabecular and cortical bone microstructure parameters separately.

**Design, Setting, and Participants:**

Using 1103 single nucleotide polymorphisms (SNPs) independently associated with estimated bone mineral density of the heel (eBMD), we developed a weighted GRS for eBMD and determined its contribution to fracture prediction beyond 2 previously developed GRSs for femur neck BMD (49 SNPs) and lumbar spine BMD (48 SNPs). Associations between these GRSs and forearm (n_cases_ = 1020; n_controls_ = 2838), hip (n_cases_ = 1123; n_controls_ = 2630) and vertebral (n_cases_ = 288; n_controls_ = 1187) fractures were evaluated in 3 Swedish cohorts. Associations between the GRSs and trabecular and cortical bone microstructure parameters (n = 426) were evaluated in the MrOS Sweden cohort.

**Results:**

We found that eBMD_GRS_ was the only significant independent predictor of forearm and vertebral fractures while both FN-BMD_GRS_ and eBMD_GRS_ were significant independent predictors of hip fractures. The eBMD_GRS_ was the major GRS contributing to prediction of trabecular bone microstructure parameters while both FN-BMD_GRS_ and eBMD_GRS_ contributed information for prediction of cortical bone microstructure parameters.

**Conclusions:**

The eBMD_GRS_ independently predicts forearm and vertebral fractures while both FN-BMD_GRS_ and eBMD_GRS_ contribute independent information for prediction of hip fractures. We propose that eBMD_GRS_ captures unique information about trabecular bone microstructure useful for prediction of forearm and vertebral fractures. These findings may facilitate personalized medicine to predict site-specific fractures as well as cortical and trabecular bone microstructure separately.

Osteoporosis is a disease characterized by low bone mass and micro-architectural deterioration of bone tissue, leading to increased risk of fragility fractures (1). Fracture risk is inversely associated with bone strength, which is dependent on bone mineral density (BMD) as well as bone quality parameters such as trabecular and cortical bone microstructure parameters (2, 3).

Ultrasound measures of the calcaneus (heel) predict fracture risk and this association partly remains after adjustment for hip BMD, suggesting that ultrasound captures unique bone property information of importance for fracture risk (4, 5). Detailed studies using three-dimensional high resolution peripheral quantitative computed tomography (HRpQCT) have revealed that both trabecular and cortical bone microstructure parameters also contribute to fracture prediction beyond two-dimensional dual-energy x-ray absorptiometry (DXA)-derived BMD (6–11). Recent data indicate that the different HRpQCT measures may predict fractures in a bone site–specific manner (12).

In the clinical setting, it is important to identify the patients at highest risk of fracture, who are most likely to benefit from osteoporosis treatment (3). Today, osteoporosis diagnosis and fracture risk prediction are based on imaging (particularly DXA) in combination with assessment of clinical risk factors; the latter are often incorporated in fracture risk prediction tools. It is likely that the genetic and environmental factors that determine fracture risk differ substantially between fractures at different bone sites with different proportions of trabecular and cortical bone. However, current available fracture risk prediction tools do not include information on trabecular or cortical bone microstructure parameters and are not designed to distinguish between differences in prediction of bone site–specific fractures (13, 14). It is possible that factors regulating trabecular bone microstructure are major determinants of vertebral fracture risk, while factors determining cortical bone parameters and risk of falls are major determinants of hip fracture risk. We propose that novel separate predictors for bone site–specific fractures as well as for cortical and trabecular bone microstructure parameters may be useful for the development of personalized medicine. A certain combination of predictors might then be used to identify high risk subjects for bone site–specific fractures and also to determine whether treatments mainly targeting trabecular or cortical bone mass should be considered.

Several bone parameters including femoral neck BMD (FN-BMD) and lumbar spine BMD (LS-BMD) measured by DXA, ultrasound measures at the calcaneus and HRpQCT-derived trabecular and cortical bone parameters are highly heritable (15–20). A meta-analysis of genome-wide association studies (GWAS) has identified 63 independent single nucleotide polymorphisms (SNPs) associated with either FN-BMD or LS-BMD (21), and a recent large scale GWAS in the UK-Biobank identified as many as 1103 independent SNPs associated with estimated BMD (eBMD) from ultrasound measures at the calcaneus (22). The identified SNPs can be used for the development of genetic risk scores (GRSs) that may be used to predict bone health-related phenotypes. Unfortunately, the number of SNPs identified by informative computed tomography (CT) measurements of trabecular and cortical bone parameters separately is low and the variance explained by these SNPs of the respective bone phenotype is marginal. Therefore, no powerful meaningful GRS for separate prediction of trabecular and cortical bone parameters can be developed (23–26).

Two previous studies have used available DXA-derived SNPs for the development of different GRSs to predict risk of fractures in independent data sets (3,27), demonstrating that these GRSs were modest predictors of fractures. Importantly, these studies did not determine the bone site-specific prediction of fracture risk. GRSs based on SNPs associated with ultrasound-derived eBMD (eBMD_GRS_) have neither been evaluated for fracture prediction in an independent data set nor for bone site-specific prediction of fracture risk (22,28).

In this study, we hypothesized that different GRSs or combination of GRSs, developed from recent well-powered GWAS on ultrasound-derived and DXA-derived relevant bone parameters, may be used for bone site–specific prediction of fracture as well as of separate prediction of trabecular and cortical bone microstructure parameters. In particular, we hypothesized that the eBMD_GRS_, developed from a high number of identified SNPs explaining a substantial part of the variance (20.3%) in eBMD at the calcaneus, may contribute information for bone site–specific fracture prediction beyond previously developed DXA-based GRSs (22). As the calcaneus is a bone with an exceptionally high trabecular bone content (29), we hypothesized that the eBMD_GRS_ might capture unique information of trabecular bone microstructure, not identified by DXA-derived GRSs, useful for prediction of fracture risk at bone sites with a relatively high proportion of trabecular bone. The unique well-powered fracture cohorts from the Umeå Fracture and Osteoporosis (UFO) study, used in the present study for comparing bone site–specific fracture prediction of the different GRSs, did not have information on traditional clinical risk factors or FN-BMD. Therefore, the aim of the present explorative study was not to determine the clinical utility of the evaluated GRSs beyond current available fracture risk prediction tools.

## Materials and Methods

### Study participants

#### UFO cohort.

The Umeå Fracture and Osteoporosis (UFO) study is a population-based study designed to identify the genetic and environmental determinants of osteoporotic fractures. This cohort is sampled from the Northern Sweden Health and Disease Study (NSHDS), a longitudinal, population-based cohort study from Northern Sweden, consisting of blood samples, and lifestyle and dietary data from approximately 100 000 unique individuals from the county of Västerbotten (approximately 255 000 inhabitants as of Dec 31, 2007) (30, 31). The UFO-hip and UFO-forearm GWAS fracture cohorts (recruited in 2008) are subcohorts of the UFO cohort, currently including approximately 5000 fracture cases and approximately 5000 controls (30). Hip and forearm fracture cases were identified by merging the NSHDS cohort with medical records and radiographic reports. From 1993 to 2008, we identified 1086 subjects with low-trauma forearm fractures and 1086 subjects with hip fractures who were also represented with a DNA sample in the biobank. A low-trauma forearm fracture was defined as a fracture resulting from a fall from a standing position or lower. Thus, forearm fractures occurring after falls from >1 m or from traffic accidents were excluded. The UFO studies have been approved by local ethical committees and informed consent was obtained from all study participants.

The UFO-forearm fracture study is a nested case-control study in which each fracture case is compared with 1 control selected from the NSHDS cohort, matched for gender and age at recruitment, comprising a total of 2172 subjects of whom 2115 passed the quality check for genotyping. All patients in the UFO-forearm fracture study completed a survey about how the forearm fracture occurred. Only fractures caused by low energy trauma were included for forearm fractures. To refine the forearm fracture phenotype in this study, 36 subjects who had another known non-forearm fracture were excluded and 101 subjects were excluded for missing height and/or weight data, resulting in a final cohort of 1978 subjects (984 forearm cases and 994 controls). The inclusion criteria for cases in the UFO-forearm GWAS fracture cohort were an age of ≥ 30 years and having a low-trauma forearm fracture as defined by medical records and/or radiograph reports. The inclusion criteria for the forearm controls were an age of ≥ 30 years with no low-trauma fracture (until year 2008) and matching for gender and age at baseline (Table 1).

**Table 1.** Characteristics of Study Participants

	UFO Hip-Fracture (n = 1873)	UFO Forearm-Fracture (n = 1978)	MrOS Sweden (n = 1880)
Age (years)	62.8 (11.7)	61.0 (6.8)	75.4 (3.2)
BMI (kg/m^2^)	25.7 (4.3)	25.5 (3.9)	26.4 (3.5)
Height (cm)	169.1 (9.1)	165.7 (7.4)	175.0 (6.5)
Weight (kg)	73.7 (14.4)	70.2 (12.5)	80.7 (12.1)
Females (n,%)	1069 (57.1)	1677 (84.8)	0 (0)
Hip fracture cases (n, %)	994 (53.1)	0 (0)	129 (6.9)
Forearm fracture cases (n, %)	0 (0)	984 (49.7)	36 (1.9)
Vertebral fractures cases (n, %)	0 (0)	0 (0)	288 (19.5*)
eBMD_GRS_	31.6 (0.6)	31.6 (0.6)	31.3 (0.6)
FN-BMD_GRS_	2.33 (0.21)	2.33 (0.21)	2.35 (0.21)
LS-BMD_GRS_	2.57 (0.22)	2.58 (0.22)	2.58 (0.23)

Values are given as mean (SD) or n (%). *Percent of patients with validated vertebral fractures was calculated based on the 1475 participants with vertebral fracture information.

The UFO-hip fracture study is a case-cohort study in which the 1086 fracture cases are compared with a set of 934 controls, comprising a total of 2020 participants, of whom 1941 passed the quality check for genotyping. Fracture patients from the larger hospital in Umeå but not from the 2 smaller hospitals in Lycksele and Skelefteå completed a survey about how the hip fracture occurred. Only fractures caused by low energy trauma were included for hip fractures for the patients in Umeå. To refine the hip fracture phenotype in this study, 19 subjects who had another known non-hip fracture were excluded and 49 subjects were excluded for missing height and/or weight data, resulting in a final cohort of 1873 participants (994 hip cases and 879 controls). The inclusion criteria for the cases in the UFO-hip GWAS fracture cohort were an age of ≥ 20 years and a hip fracture defined by medical records and/or radiograph reports. Subjects with a previous known low-trauma forearm fracture were excluded. The UFO-hip GWAS fracture controls were drawn from the controls of a previous GWAS study of glioma (32) (Table 1).

### MrOS Sweden

The Osteoporotic Fractures in Men (MrOS) study is a multicenter, prospective study including older men in Sweden, Hong Kong, and the United States. The MrOS Sweden cohort consists of 3 sub-cohorts from 3 Swedish cities (n  =  1005 in Malmö, n  =  1010 in Gothenburg, and n  =  999 in Uppsala). Study participants (men aged 69 to 81 years) were randomly selected through national population registers, contacted, and asked to participate. A total of 45% of the subjects who were contacted participated in the study. To be eligible for the study, the participants had to be able to walk without assistance, provide self-reported data, and sign an informed consent (33). In this study, we included 1880 participants (953 from Gothenburg and 927 participants from Malmö) with available genetic data that passed the quality check for genotyping (Table 1). Participants in the MrOS Sweden cohort were followed for up to 10 years after the baseline examination. Fracture evaluation was done by searching digital x-ray archives and matching them with MrOS Sweden participants using unique personal identification number, which all Swedish citizens have. All reported fractures after baseline were confirmed by physician review of radiology report. Fractures with ICD10 code S72.0, S72.1, or S72.2 were classified as hip fractures while ICD10 code S52.5 and S52.6 were classified as forearm fractures (Table 1). Vertebral fractures were identified using thoracic spine x-ray at up to 3 occasions (at baseline, after 3 years and after 5 years). The radiographs were evaluated for radiographic vertebral fractures by an expert radiologist using a modified semiquantitative method developed by Genant and colleagues (34, 35). Individuals with a radiographic vertebral fracture at any of the occasions were classified as fracture cases while individuals with at least 1 x-ray and no vertebral fracture identified were classified as non-cases. Individuals without any thoracic spine x-ray were excluded from the analyses of radiographic vertebral fractures (Table 1). No information about the underlying cause of fracture is available for MrOS Sweden. The study was approved by the ethics committees at the Universities of Gothenburg, Lund, and Uppsala and informed consent was obtained from all study participants.

## Genotyping

### UFO cohort

#### UFO-forearm fracture cohort.

Genotyping of the forearm fracture cohort was performed using Illumina Omni express arrays. Genotypes were called using the BeadStudio calling algorithm. The sample quality control exclusion criteria were a sample call rate < 97.5%, gender discrepancy with genetic data from X-linked markers, excess autosomal heterozygosity > 0.33 (false discovery rate [FDR] < 0.1%), duplicates and/or first-degree relatives identified using identical-by-state (IBS) probabilities (>97%), ethnic outliers (3 standard deviations [SD] away from the population mean) using multidimensional scaling analysis with 4 principal components. Genotypes from 2115 individuals (1055 controls and 1060 cases) passed the sample quality control.

#### UFO hip fracture cohort.

Genotyping of the hip fracture cohort was performed using Illumina HumanHap660 arrays. Genotypes were called using the BeadStudio calling algorithm. The sample quality control exclusion criteria were a sample call rate < 97.5%, gender discrepancy with genetic data from X-linked markers, excess autosomal heterozygosity > 0.33 (~FDR < 0.1%), duplicates and/or first degree relatives identified using IBS probabilities (> 97%), ethnic outliers (3 SD away from the population mean) using multidimensional scaling analysis with 4 principal components. Genotypes from 1941 individuals (891 controls and 1050 cases) passed the sample quality control.

### MrOS Sweden

#### Gothenburg part.

Genotyping, imputation, and quality controls were performed using the Illumina HumanOmni1_Quad_v1-0 B array. Genotypes were called using the Illumina’s BeadStudio calling algorithm. The sample quality control exclusion criteria were a sample call rate < 97%, excessive autosomal heterozygosity, first- and second-degree relatives, genotypic sex mismatch using X and Y chromosome probe intensities, and gross chromosome abnormalities. Genotyped SNPs with GenTrain scores < 0.6, cluster separation scores < 0.4, call rates < 97%, or minor allele frequency < 0.01 were excluded. Also, autosomal SNPs with Hardy-Weinberg Equilibrium *P* value < 10^−4^ were excluded and genotype clusters for SNPs on chrX, chrY, chrXY, and chrMT were reviewed manually. 714 543 autosomal SNPs passed quality control. Genotypes from 953 individuals passed the sample quality control.

#### Malmö part.

Genotyping and quality controls were performed using the HumanOmniExpress-12v1_B build 36. The sample quality control exclusion criteria were sample call rate < 97.5%, missing data, excessive autosomal heterozygosity, familiar relationship (1 sample excluded), genotypic sex mismatch, non-Caucasian ethnicity, and gross chromosome abnormalities. SNPs with call rates < 95% were excluded. 725 409 autosomal SNPs passed quality control. Genotypes from 927 individuals passed the sample quality control.

### Imputation

For all cohorts, genotypes were imputed to the Haplotype Reference Consortium release 1.1 reference panel (36) yielding dosages for all SNPs, that is, continuous estimates of the number of risk alleles. The imputation was done separately for each cohort. Both pre-phasing and imputation were done using Sanger Imputation Service. SNPs with no variance or low imputation quality (< 0.3) were excluded. The HRC.r1 release consists of 64 940 haplotypes and a total of 40 405 505 variants of predominantly European ancestry.

### Three different genetic risk scores

We defined 3 GRSs (FN-BMD_GRS_, LS-BMD_GRS_, and eBMD_GRS_) based on previously identified SNPs associated with the corresponding BMD measure in large-scale GWAS (21,22). A total of 1103 independent SNPs have been identified to associate with eBMD (22). In MrOS Sweden and the UFO cohorts, 1083 and 1085 SNPs, respectively, out of the 1103 SNPs had high-quality imputed dosage information available and were used for the calculation of the eBMD_GRS_. The GWAS results, including the effect sizes for the 1103 eBMD SNPs used in this study, are publicly available from Supplementary Table 2 in the original publication (22). Out of the 63 independent SNPs identified in the GWAS on FN-BMD and LS-BMD (21), 49 SNPs were associated with FN-BMD (*P* < 5 × 10^–8^) and included in the FN-BMD_GRS_ while 48 of the 63 SNPs were associated with LS-BMD (*P* value < 5 × 10^–8^) and included in the LS-BMD_GRS_. All these 63 SNPs were imputed with high quality in UFO while 1 SNP had low imputation quality in MrOS Sweden and was excluded. The GWAS results, including the effect sizes for the 49 SNPs associated with FN-BMD and the 48 SNPs associated with LS-BMD are publicly available from Supplementary Tables 4A and 4B in the original publication (21). There is an overlap between the SNPs in the eBMD_GRS_ and the 2 DXA-derived GRSs. A total of 27 of the SNPs in FN-BMD_GRS_ and 28 of the SNPs in LS-BMD_GRS_ are represented in the eBMD_GRS_ by either the same SNP or a SNP with an *R*^*2*^ ≥ 0.8. For each individual, the GRSs were defined as the weighted sum of SNP dosages, where SNP effects from the corresponding BMD GWAS were used as weights. The GRSs were standardized to have a mean of zero and SD of 1.

### Bone measures

#### Estimated BMD (eBMD) using ultrasound.

Speed of sound (SOS) and bone ultrasound attenuation (BUA) were measured by quantitative ultrasound (Hologic Sahara, Waltham, MA) at the left calcaneus in MrOS Sweden participants (37). The eBMD was then calculated as a linear combination of SOS and BUA using the following algorithm eBMD = 0.0025926 × (BUA + SOS) − 3.687) (38). Thus, eBMD is an indirectly measured BMD (Table 2). The eBMD is not used for osteoporosis diagnosis or fracture prediction in the clinical context, but some studies have shown that not only DXA-derived BMD but also ultrasound-derived eBMD is strongly associated with fracture risk (39, 40).

**Table 2.** Bone Parameters in MrOS Sweden

Ultrasound (n = 1880)	
Estimated BMD of Calcaneus (g/cm^2^)	0.53 (0.15)
**DXA (n = 1880)**	
Femur Neck BMD (g/cm^2^)	0.83 (0.13)
Lumbar Spine BMD (g/cm^2^)	1.13 (0.20)
**HR pQCT of Distal Radius (n = 426)**	
Failure Load (N)	3822 (827)
**Trabecular parameters**	
Trabecular vBMD (mg/cm^3^)	165 (38)
Trabecular Number (1/mm)	2.1 (0.3)
Trabecular Thickness (µm)	67 (12)
**Cortical prameters**	
Cortical Area (mm^2^)	53.2 (18.4)
Cortical vBMD (mg/cm^3^)	886 (53)
Cortical Porosity (%)	4.3 (1.7)

Values are given as mean (SD).

Abbreviations: estimated BMD, estimated bone mineral density analysed by ultrasound; femur neck BMD, femoral neck bone mineral density analysed by dual energy absorptiometry (DXA); HRpQCT, high resolution peripheral quantitative computed tomography; lumbar spine BMD, lumbar spine bone mineral density analysed by DXA; vBMD, volumetric bone mineral density.

#### Areal BMDs using DXA.

Areal BMDs (g/cm^2^) of the femoral neck (FN-BMD) and lumbar spine (LS-BMD; L1–L4) were assessed using the Lunar Prodigy DXA (GE Lunar Corp., Madison, WI) for the subjects investigated in MrOS Sweden/Malmö, or the Hologic DXA Hologic QDR 4500/A‐Delphi (Hologic, Whaltman, MA) for subjects investigated in MrOS Sweden/Gothenburg. The coefficient of variations for the areal BMD measurements ranged from 0.5% to 3%, depending on application. To be able to use DXA measurements performed with equipment from the 2 different manufacturers, standardized BMD was calculated for these bone sites as previously described (33, 41–43) (Table 2).

#### High resolution peripheral quantitative computed tomography (HRpQCT).

Volumetric bone density (vBMD) and bone microarchitecture were assessed at the ultradistal radius with XtremeCT scanners (Scanco Medical, Switzerland), which were used by operators trained by the manufacturer (11). Scans were acquired with a nominal isotropic voxel size of 82 µm^3^. Scanning was done on the nondominant forearm. If a participant reported previous extremity fracture or had metal in the scan region, the contralateral extremity was examined. Anteroposterior scout views were used to place a reference line on the distal radial joint surface (44). The radial scan region was 9 mm in length (110 slices) and offset proximally to the reference line by 9.5 mm. Scanning of a quality control phantom limb containing rods of hydroxyapatite at densities of 0, 100, 200, 400, and 800 mg hydroxyapatite per cm^3^ was done daily to monitor long-term stability of the system. Scans were graded with a 5-point motion artefact scale (1 = none, 2 = minor, 3 = moderate, 4 = severe, and 5 = extreme) (45). For density measures, scans with movement artefacts graded 1 to 4 were retained, and for microarchitecture measures, those graded 1 to 3 were retained. A standard analysis program (Scanco software version 6.0) was used to assess total cross-sectional area, density, trabecular density, and trabecular microarchitecture, and a semi-automated cortical bone segmentation technique was used to assess cortical density and cortical microarchitecture (46). All bone measures were standardized to have a mean of zero and SD of 1 (Table 2).

### Statistical analyses

Associations between the 3 GRSs and risk of forearm, hip, and vertebral fractures were first evaluated in separate logistic regression models, adjusted for age, sex, height, weight, and MrOS site when applicable. Differences between log odds for the separate GRSs were tested for significance using a z-test. Next, independent associations between the 3 GRSs and risk of forearm, hip, and vertebral fractures were evaluated in combined logistic regression models. The independently associated GRSs were selected by forward stepwise selection in logistic regression models starting from a fixed base model including age, sex, height, weight, and MrOS site. We then validated that the final models, including either 1 or 2 independently associated GRSs, also resulted in the lowest Akaike information criterion (AIC) for fracture prediction. The analyses were done separately for the UFO-forearm, the UFO-hip and the MrOS Sweden cohorts and the cohort–specific effects were then combined using inverse variance weighted meta-analysis. Association between the 3 GRSs and different bone parameters in MrOS Sweden were first evaluated in separate linear regression models, adjusted for age, sex, height, weight, and MrOS site. Differences between bone measure associations for the separate GRSs were tested for significance using a z-test. Next, independent association between the 3 GRSs and different bone parameters in MrOS Sweden were evaluated using stepwise selection in combined linear regression models, adjusted for age, sex, height, weight, and MrOS site. All analyses were performed using R version 3.4.2. Area under the receiver operating characteristic curve (AUC) and corresponding confidence intervals were calculated using the roc.test function in the pROC R-package (47).

## Results

### The ultrasound-based genetic risk score eBMD_GRS_ displays modest correlations with the DXA-based genetic risk scores FN-BMD_GRS_ and LS-BMD_GRS_

The means and SDs of the 3 bone-related GRSs, including the heel ultrasound-based eBMD_GRS_ and the DXA-based FN-BMD_GRS_ and LS-BMD_GRS_, were similar in the 3 evaluated Swedish cohorts, UFO hip fracture, UFO forearm fracture, and MrOS Sweden (Table 1). To determine if these 3 GRSs might have the potential to contribute independent information for prediction of bone health–related parameters, we first evaluated their intercorrelations in the 3 clinical cohorts used in the present study (Table 3). The FN-BMD_GRS_ explained as much as 53.5% to 55% of the variance of the other DXA-based genetic risk score, LS-BMD_GRS_, in the 3 cohorts. In contrast, the variances in the 2 DXA-based risk scores explained by the newly-developed ultrasound-based eBMD_GRS_ were rather modest: FN-BMD_GRS_ (9.2% to 13.5%) and LS-BMD_GRS_ (12.6% to 14.8%) (Table 3). Thus, the new eBMD_GRS_ might contribute substantial independent information, beyond the 2 previously evaluated DXA-based GRSs, for prediction of different bone-related parameters. We next confirmed in the MrOS Sweden cohort that the different evaluated GRSs were associated with the underlying bone phenotype used for the development of the respective GRS (with a high GRS indicating a low value of the bone parameter). As expected, the eBMD_GRS_ was significantly inversely associated with eBMD (variance explained *R*^2^ = 17.0%; *P* = 8.5 × 10^−68^), the FN-BMD_GRS_ was significantly inversely associated with FN-BMD (*R*^2^ = 3.9%; *P* = 5.0 × 10^−21^) and the LS-BMD_GRS_ was significantly inversely associated with LS-BMD (*R*^2^ = 4.6%; *P* = 1.2 × 10^−23^) (Table 4).

**Table 3.** Cross Tab of Variance Explained (*R*^2^) for the 3 Genetic Risk Scores

	FN-BMD_GRS_	LS-BMD_GRS_
**UFO Hip-Fracture**		
eBMD_GRS_	12.2%	13.3%
FN-BMD_GRS_		53.5%
**UFO Forearm-Fracture**		
eBMD_GRS_	13.5%	14.8%
FN-BMD_GRS_		54.8%
**MrOS-Sweden**		
eBMD_GRS_	9.2%	12.6%
FN-BMD_GRS_		55.0%

Abbreviations: eBMD_GRS_, genetic risk score for estimated bone mineral density of the heel; FN-BMD_GRS_, genetic risk score for bone mineral density in femur neck; LS-BMD_GRS_, genetic risk score for bone mineral density in lumbar spine.

**Table 4.** Association Between 3 Genetic Risk Scores (GRSs) and Different Bone Parameters in MrOS Sweden, Evaluated in Separate Linear Regression Models

		eBMD_GRS_				FN-BMD_GRS_					LS-BMD_GRS_				
	N	Beta	SE	*P*	*R* ^2^	Beta	SE	*P*	*R* ^2^		Beta	SE	*P*	*R* ^2^	
**Ultrasound Calcaneus**															
eBMD	1567	−0.41	0.02	**8.5E-68**	17.0%	−0.15	0.02	**1.0E-09**	2.3%	*	−0.16	0.02	**1.0E-10**	2.5%	*
**DXA**															
FN-BMD	1861	−0.21	0.02	**1.0E-24**	4.6%	−0.20	0.02	**5.0E-21**	3.9%		−0.15	0.02	**3.1E-12**	2.2%	*
LS-BMD	1865	−0.23	0.02	**3.1E-27**	5.4%	−0.17	0.02	**2.5E-16**	3.1%		−0.21	0.02	**1.2E-23**	4.6%	
**HRpQCT of Distal Radius**															
Failure Load	354	−0.38	0.05	**7.0E-14**	12.6%	−0.24	0.05	**1.4E-06**	5.5%	*	−0.22	0.05	**3.1E-05**	4.1%	*
**Trabecular parameters**															
vBMD	403	−0.39	0.05	**4.0E-15**	13.8%	−0.24	0.05	**1.2E-06**	5.5%	*	−0.26	0.05	**9.0E-07**	5.6%	
Trabecular Number	357	−0.33	0.05	**1.2E-09**	9.2%	−0.20	0.05	**9.2E-05**	3.9%		−0.24	0.05	**9.2E-06**	5.0%	
Trabecular Thickness	357	−0.31	0.05	**9.7E-09**	8.2%	−0.15	0.05	**4.3E-03**	2.1%	*	−0.14	0.05	**1.2E-02**	1.6%	*
**Cortical parameters**															
Area	426	−0.24	0.05	**3.8E-07**	5.3%	−0.22	0.05	**1.6E-06**	4.8%		−0.23	0.05	**2.2E-06**	4.6%	
vBMD	397	−0.20	0.05	**3.9E-05**	3.8%	−0.22	0.05	**7.4E-06**	4.5%		−0.20	0.05	**1.3E-04**	3.3%	
Porosity	352	0.04	0.06	4.7E-01	0.1%	0.05	0.05	3.2E-01	0.3%		0.05	0.06	4.1E-01	0.2%	

The models for ultrasound and dual energy absorptiometry (DXA) parameters, available both in the Gothenburg and the Malmö cohort of MrOS Sweden, are adjusted for age, height, weight, and MrOS site. The models for high resolution peripheral quantitative computed tomography (HRpQCT)-derived parameters in the distal radius, available only in the Gothenburg part of MrOS Sweden, are adjusted for age, height, and weight. Betas are expressed as SD change in bone parameter per SD increase in GRS.

Abbreviations: eBMD, estimated bone mineral density analysed by ultrasound; FN-BMD, femoral neck bone mineral density analysed by DXA; LS-BMD, lumbar spine bone mineral density analysed by DXA; vBMD, volumetric bone mineral density.

* *P* < 0.05 vs eBMD_GRS_

### The eBMD_GRS_ is an independent predictor of vertebral and forearm fractures, while both the FN-BMD_GRS_ and the eBMD_GRS_ contribute to hip fracture prediction

**Figure 1.**
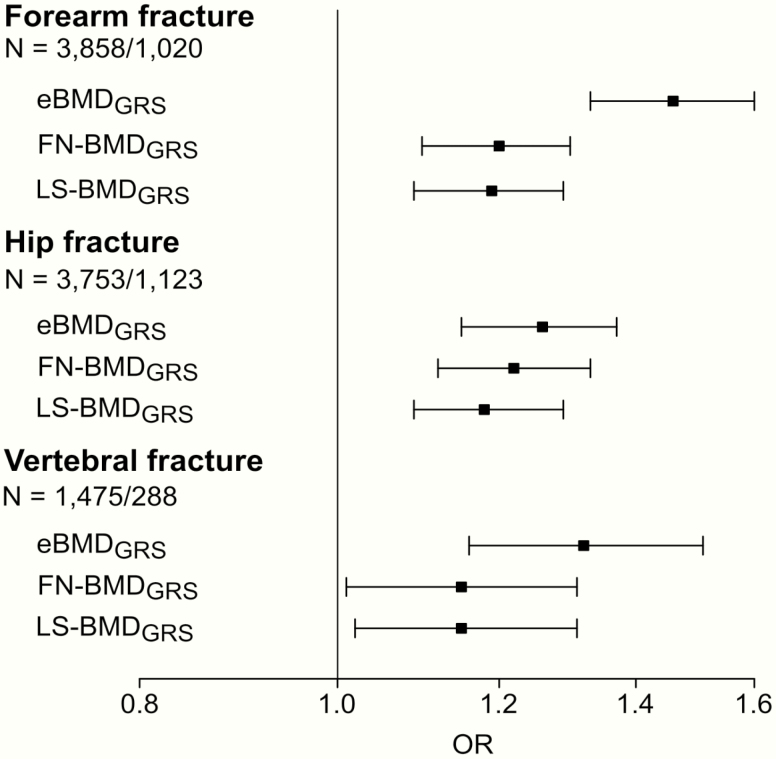
Associations between 3 GRSs and risk of forearm, hip, and vertebral fractures, evaluated in separate logistic regression models. Models are adjusted for age, sex, height, weight, and MrOS site. Odds ratios (OR) and 95% confidence intervals given per SD increase of the genetic risk score (GRS) from inverse variance weighted meta-analysis of significant independent associations. The association between the eBMD_GRS_ and forearm fractures was significantly stronger than the corresponding associations for the FN-BMD_GRS_ (*P* = 0.002) and the LS-BMD_GRS_ (*P* = 0.001). N = total number of subjects/fracture cases. Abbreviations: eBMD, estimated bone mineral density analysed by ultrasound; FN-BMD, femoral neck bone mineral density analysed by dual-energy absorptiometry; LS-BMD, lumbar spine bone mineral density analysed by dual-energy absorptiometry.

**Figure 2.**
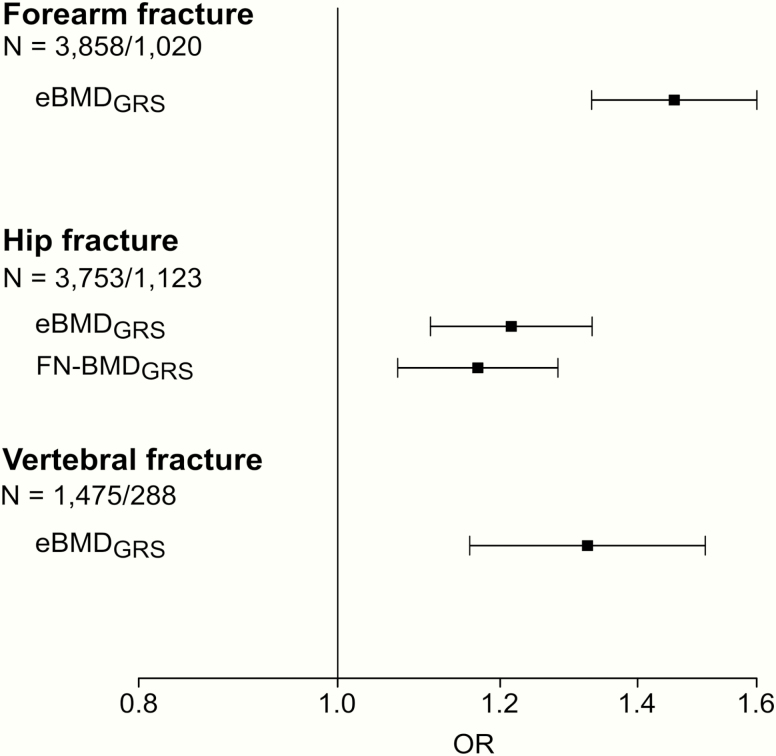
GRSs independently associated with risk of forearm, hip and vertebral fractures. The independently associated genetic risk scores (GRSs) were selected by forward stepwise selection in logistic regression models starting from a fixed base model including age, sex, height, weight, and MrOS site. We then validated that the final models, including either 1 or 2 independently associated GRSs, also resulted in the lowest AIC. Odds ratios (OR) and 95% confidence intervals given per SD increase in GRS from inverse variance weighted meta-analysis of significant independent associations. N = total number of subjects/fracture cases. Abbreviations: eBMD, estimated bone mineral density analysed by ultrasound; FN-BMD, femoral neck bone mineral density analysed by dual-energy absorptiometry.

We next compared the separate and combined performances of the 3 bone-related GRSs for prediction of vertebral fractures, forearm, and hip fractures. Associations between the 3 GRSs and risk of forearm, hip, and vertebral fractures were first evaluated in separate logistic regression models. All 3 GRSs were significantly directly associated with fracture risk at all 3 bone sites (Fig 1). For forearm fractures and vertebral fractures, the effect sizes, expressed as odds ratio (OR) per SD increase in GRS, were more pronounced for the eBMD_GRS_ compared with the 2 DXA-based GRSs, while for hip fractures the effect sizes were rather similar for the FN-BMD_GRS_ and the eBMD_GRS_ (Fig 1). AUCs for fracture discrimination for the GRSs in each of the included cohorts are given in Table 5. In general, these AUC data support the notion that the eBMD_GRS_ is the most informative GRS for prediction of wrist and vertebral fractures, while the AUCs of the eBMD_GRS_ and the FN-BMD_GRS_ are of similar magnitude for hip fracture discrimination. As the GRSs were correlated with each other, we next evaluated the independent associations between the 3 GRSs and risk of fractures, using stepwise selection in combined logistic regression models (Fig 2). In these analyses, we observed that the eBMD_GRS_ was the only independent predictor of forearm (OR [per SD increase] 1.46; 95% CI, 1.33-1.60) and vertebral fractures (OR 1.32; 95% CI, 1.16-1.51). In contrast, both the FN-BMD_GRS_ (OR 1.17; 95% CI, 1.07-1.28) and the eBMD_GRS_ (OR 1.21; 95% CI, 1.11-1.33) contributed independent information for prediction of hip fractures.

**Table 5.** Area Under the ROC Curve (AUC) for 3 Genetic Risk Scores (GRSs) for Prediction of Risk of Forearm, Hip, and Vertebral Fractures, Evaluated in Separate Cohorts

	UFO Hip-Fracture N = 1873			UFO Forearm Fracture N = 1978			MrOS Sweden N = 1880*		
	AUC	95% CI	N cases	AUC	95% CI	N cases	AUC	95% CI	N cases
**Hip fracture**			994						129
eBMD_GRS_	0.56	(0.54, 0.59)					0.56	(0.51, 0.61)	
FN-BMD_GRS_	0.56	(0.53, 0.58)					0.57	(0.52, 0.62)	
LS-BMD_GRS_	0.55	(0.52, 0.58)					0.56	(0.50, 0.62)	
**Forearm fracture**						984			36
eBMD_GRS_				0.60	(0.57, 0.62)		0.66	(0.58, 0.75)	
FN-BMD_GRS_				0.55	(0.52, 0.57)		0.60	(0.50, 0.70)	
LS-BMD_GRS_				0.55	(0.53, 0.58)		0.58	(0.47, 0.69)	
**Vertebral fracture**									288
eBMD_GRS_							0.57	(0.53, 0.61)	
FN-BMD_GRS_							0.55	(0.51, 0.59)	
LS-BMD_GRS_							0.54	(0.51, 0.58)	

Area under the ROC curve (AUC) and 95% confidence intervals (CI) are given for each GRS in separate unadjusted models. N = number of participants. N cases = number of fracture cases.

Abbreviations: eBMD_GRS_, genetic risk score for estimated bone mineral density of the heel; FN-BMD_GRS_, genetic risk score for bone mineral density in femur neck; LS-BMD_GRS_, genetic risk score for bone mineral density in lumbar spine.

*For vertebral fractures, only N = 1475 participants were included.

We next performed an explorative subanalysis dividing up both the eBMD_GRS_ and the FN-BMD_GRS_ into 1 GRS that included only the common SNPs (27 SNPs) between the eBMD_GRS_ and FN-BMD_GRS_ and 1 GRS containing only the SNPs specific for either the FN-BMD_GRS_ (22 SNPs) or the eBMD_GRS_ (1076 SNPs). This results in 6 different GRSs (eBMD_GRS_, CommonSNP-eBMD_GRS_, SpecificSNP-eBMD_GRS_, FN-BMD_GRS_ and CommonSNP-FNBMD_GRS_, SpecificSNP-FNBMD_GRS_) (Table 6) for comparison of their associations with fracture risk at different bone sites. The CommonSNP-eBMD_GRS_ and the CommonSNP-FNBMD_GRS_, both including the same common 27 SNPs, displayed very similar patterns of association with fracture risk at all 3 bone sites (Table 6) and these associations were also very similar to the associations for the FN-BMD_GRS_ for fractures at all 3 bone sites. In contrast, the eBMD_GRS_ displayed stronger associations with forearm and vertebral fractures, but not with hip fractures, compared with the corresponding associations for the 2 Common SNP GRSs. Interestingly, the SpecificSNP-eBMD_GRS_ demonstrated very similar fracture prediction as the eBMD_GRS_ for fractures at all 3 bone sites while the SpecificSNP-FNBMD_GRS_ was only associated with risk of hip fractures. These exploratory subanalyses support the notion that a substantial part of the better performance of the eBMD_GRS_ compared with the FN-BMD_GRS_ is dependent on the larger number of SNPs and variance explained by the eBMD_GRS_. Yet, the specific eBMD SNPs seem to add unique information not captured by the FN-BMD_GRS_ for prediction of forearm and vertebral fractures.

**Table 6.** Comparison of Fracture Risk Associations Using Different SNP Selections for Genetic Risk Scores (GRS)

				Forearm Fracture N = 3858/1020			Hip Fracture N = 3753/1123			Vertebral Fracture N = 1475/288		
	Selection of SNPs	Number of SNPs	Variance (% explained of the underlying bone phenotype in MrOS Sweden)	OR	95% CI	*P*	OR	95% CI	*P*	OR	95% CI	*P*
eBMD_GRS_	All SNPs	1103	17.0%	1.46	(1.33, 1.60)	**1.2E-16**	1.26	(1.15, 1.37)	**1.4E-07**	1.32	(1.16, 1.51)	**4.5E-05**
FN-BMD_GRS_	All SNPs	49	3.9%	1.20	(1.10, 1.30)	**5.2E-05**	1.22	(1.12, 1.33)	**3.3E-06**	1.15	(1.01, 1.31)	**3.1E-02**
CommonSNP-eBMD_GRS_	Common SNPs	27	3.3%	1.27	(1.16, 1.38)	**1.0E-07**	1.20	(1.11, 1.31)	**6.2E-06**	1.13	(0.99, 1.29)	6.0E-02
CommonSNP-FNBMD_GRS_	Common SNPs	27	1.8%	1.24	(1.14, 1.36)	**8.5E-07**	1.20	(1.11, 1.30)	**1.0E-05**	1.14	(1.00, 1.30)	**4.3E-02**
SpecificSNP-eBMD_GRS_	Specific SNPs	1076	15.7%	1.40	(1.28, 1.53)	**9.4E-14**	1.27	(1.17, 1.38)	**3.8E-08**	1.30	(1.14, 1.49)	**9.3E-05**
SpecificSNP-FNBMD_GRS_	Specific SNPs	22	2.2%	1.02	(0.94, 1.11)	6.0E-01	1.23	(1.13, 1.34)	**1.5E-06**	1.07	(0.94, 1.22)	2.9E-01

Both eBMD_GRS_ and the FN-BMD_GRS_ were divided into 1 GRS including only the common SNPs (27 SNPs) between the eBMD_GRS_ and FN-BMD_GRS_ and 1 GRS including only SNPs specific for either the FN-BMD_GRS_ (22 SNPs) or the eBMD_GRS_ (1076 SNPs). This results in 6 different GRSs (eBMD_GRS_, CommonSNP-eBMD_GRS_, SpecificSNP-eBMD_GRS_, FN-BMD_GRS_ and CommonSNP-FNBMD_GRS_, SpecificSNP-FNBMD_GRS_. The table displays odds ratios (OR) and 95% confidence intervals (CI) for fracture risk for the 6 different GRSs and the 3 different fracture types.

Abbreviations: eBMD_GRS_, genetic risk score for estimated bone mineral density of the heel; FN-BMD_GRS_, genetic risk score for bone mineral density in femur neck; SNP, single nucleotide polymorphism.

### The eBMD_GRS_ is the major predictor of trabecular bone microstructure parameters, while both the FN-BMD_GRS_ and the eBMD_GRS_ contribute independent information for prediction of cortical bone area and cortical density

#### eBMD and DXA-based BMD.

We first compared the associations between the 3 GRSs and the different underlying ultrasound and DXA-based bone parameters in MrOS Sweden, evaluated in separate models. We observed that for eBMD, the eBMD_GRS_ explained substantially more of the variance than what the 2 DXA-based GRSs did (Table 4). In contrast, the FN-BMD_GRS_ explained the variance of FN-BMD similarly well as the eBMD_GRS_ did and the LS-BMD_GRS_ explained the variance of LS-BMD similarly well as the eBMD_GRS_ did. When evaluated using stepwise selection in combined linear regression models, only the eBMD_GRS_ was independently associated with eBMD while both the FN-BMD_GRS_ and the eBMD_GRS_ were independently associated with FN-BMD, and both the LS-BMD_GRS_ and the eBMD_GRS_ were independently associated with LS-BMD (Table 7). Thus, the eBMD_GRS_ contributes to the prediction of the DXA-based BMDs beyond the site-specific GRSs while the DXA-based GRSs do not contribute to the prediction of eBMD beyond the eBMD_GRS_.

**Table 7.** Association Between 3 Genetic Risk Scores (GRSs) and Different Bone Parameters in MrOS Sweden, Evaluated Using Stepwise Selection in Combined Linear Regression Models

		eBMD_GRS_			FN-BMD_GRS_			LS-BMD_GRS_		
	N	Beta	SE	*P*	Beta	SE	*P*	Beta	SE	*P*
**Ultrasound Calcaneus**										
eBMD	1567	−0.41	0.02	**8.5E-68**						
**DXA**										
FN-BMD	1861	−0.17	0.02	**3.0E-15**	−0.15	0.02	**1.6E-11**			
LS-BMD	1865	−0.17	0.02	**3.4E-15**				−0.15	0.02	**1.4E-11**
**HRpQCT of Distal Radius**										
Failure Load	354	−0.34	0.05	**3.9E-11**	−0.16	0.05	**9.2E-04**			
**Trabecular parameters**										
vBMD	403	−0.35	0.05	**4.3E-12**	−0.15	0.05	**1.6E-03**			
Trabecular Number	357	−0.29	0.05	**1.1E-07**	−0.13	0.05	**9.9E-03**			
Trabecular Thickness	357	−0.31	0.05	**9.7E-09**						
**Cortical parameters**										
Area	426	−0.19	0.05	**4.9E-05**	−0.17	0.05	**2.2E-04**			
vBMD	397	−0.16	0.05	**1.9E-03**	−0.18	0.05	**3.5E-04**			

The models for ultrasound and dual energy absorptiometry (DXA) parameters, available both in the Gothenburg and the Malmö cohort of MrOS Sweden, are adjusted for age, height, weight, and MrOS site. The models for high resolution peripheral quantitative computed tomography (HRpQCT)-derived parameters in the distal radius, available only in the Gothenburg part of MrOS Sweden, are adjusted for age, height, and weight. Betas are expressed as SD change in bone parameter per SD increase in GRS of significant independent associations.

Abbreviations: eBMD, estimated bone mineral density analysed by ultrasound; FN-BMD, femoral neck bone mineral density analysed by DXA; LS-BMD, lumbar spine bone mineral density analysed by DXA; vBMD, volumetric bone mineral density.

#### Trabecular and cortical bone microstructure parameters.

We next hypothesized that the different GRSs might predict cortical and trabecular bone microstructure parameters differentially. The separate and combined associations between the 3 GRSs and bone microstructure parameters were evaluated using HRpQCT measurements at the distal radius (the bone site for forearm fractures) available in the MrOS Sweden cohort.

**Figure 3.**
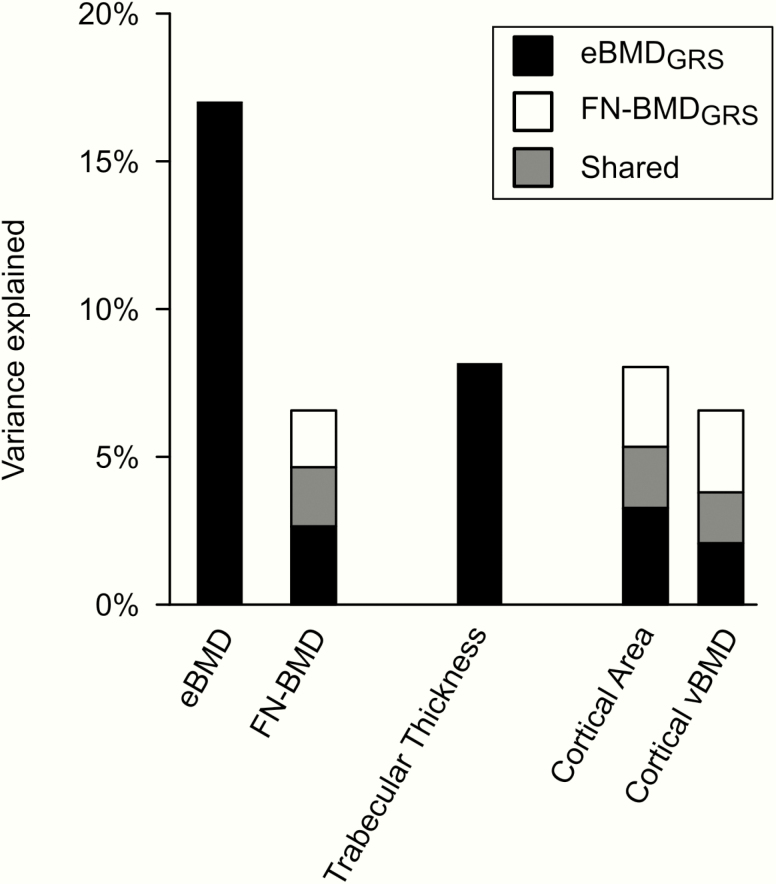
The variance explained (*R*^2^) of different bone parameters in MrOS Sweden by eBMD_GRS_ and FN-BMD_GRS_ evaluated using stepwise selection in combined linear regression analyses. Black bars = variance explained independently by eBMD_GRS_. White bar = variance explained independently by FN-BMD_GRS_. Grey bar = variance explained shared by eBMD_GRS_ and FN-BMD_GRS_. Trabecular thickness, cortical bone area and cortical volumetric BMD (cortical vBMD) were analysed by high resolution peripheral quantitative computed tomography. Abbreviations: eBMD, estimated bone mineral density analysed by ultrasound; FN-BMD, femoral neck bone mineral density analysed by dual-energy absorptiometry; GRS, genetic risk score.

When comparing the separate association between the 3 GRSs and different bone microstructure parameters, we observed that the eBMD_GRS_ was the main predictor of the trabecular bone parameters vBMD, trabecular number, and trabecular thickness, as well as of the overall bone strength-related parameter failure load. In contrast, the DXA-based FN-BMD_GRS_ predicted the cortical bone parameters cortical area and cortical volumetric BMD similarly to the eBMD_GRS_ (Table 4). Interestingly, none of the evaluated GRSs displayed any tendency of association with cortical porosity. When evaluated using stepwise selection in combined linear regression models, eBMD_GRS_ was the only independent predictor of trabecular thickness (*R*^2^ = 8.2%) and the major predictor of trabecular vBMD (*R*^2^ = 13.8%), trabecular number (*R*^2^ = 9.2%), and failure load (*R*^2^ = 12.6%) (Table 7 and Fig 3). In contrast, both the FN-BMD_GRS_ and the eBMD_GRS_ contributed independently and approximately equally to the prediction of the cortical bone parameters cortical bone area and cortical vBMD (Table 7).

## Discussion

**Figure 4.**
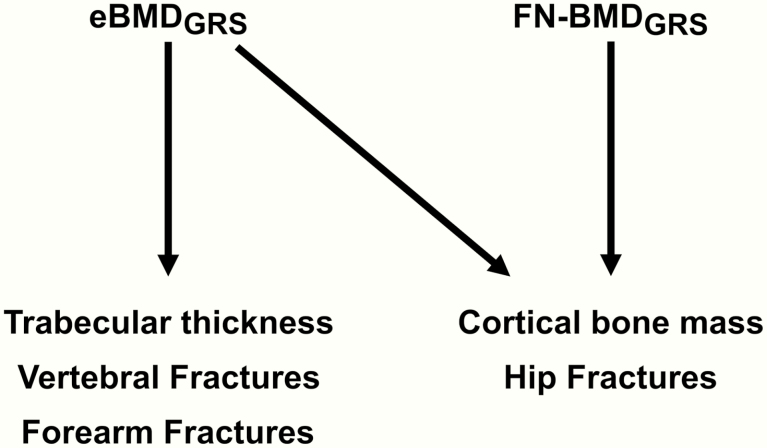
Summary of the separate and combined prediction of fracture types and trabecular and cortical bone microstructure by eBMD_GRS_ and FN-BMD_GRS_. eBMD_GRS_ but not FN-BMD_GRS_ independently predicted trabecular bone thickness as well as vertebral and forearm fractures. In contrast, both FN-BMD_GRS_ and eBMD_GRS_ independently predicted cortical bone mass parameters (cortical area and cortical thickness) and hip fractures. We propose that eBMD_GRS_ captures unique information of trabecular bone microstructure useful for the prediction of forearm and vertebral fractures. In contrast, both FN-BMD_GRS_ and eBMD_GRS_ capture unique information of cortical bone mass useful for the prediction of hip fractures. Abbreviations: eBMD, estimated bone mineral density analysed by ultrasound; FN-BMD, femoral neck bone mineral density analysed by dual-energy absorptiometry.

The differences in genetic contribution to bone site–specific fracture risk and to trabecular versus cortical bone microstructure parameters are poorly investigated. In this study, we hypothesized that different GRSs or combinations of GRSs, developed from recent well-powered GWAS on ultrasound-derived and DXA-derived relevant bone parameters, may be used for the separate prediction of fractures at different bone sites, including forearm, vertebral, and hip fractures, as well as for separate prediction of trabecular and cortical bone microstructure parameters. We demonstrate that the ultrasound-based calcaneus eBMD_GRS_ captures unique information of trabecular bone microstructure parameters as well as of risk of vertebral and forearm fractures, while both the DXA-based FN-BMD and the eBMD_GRS_ capture unique information of cortical bone mass and hip fracture risk (Fig 4).

Two previous studies have shown that fracture risk prediction is modest when only using available DXA-derived GRSs (3, 27). The bone site-specific prediction of fracture risk was, however, not evaluated in these studies. In addition, no previous study has evaluated the impact of any bone-related GRS for cortical and trabecular bone microstructure parameters. In the present study we observed that the ultrasound-based eBMD_GRS_ displayed modest correlations with the 2 DXA-based GRSs, demonstrating that the eBMD_GRS_ might contribute with independent information beyond the information from DXA-derived GRSs for prediction of bone health–related parameters. Besides contributing unique bone property information not captured by the DXA-derived GRSs, the eBMD_GRS_ explained approximately 5 times more of the variance of the underlying bone phenotype than the 2 evaluated DXA-based GRSs. The latter was most likely the result of the exceptional large UK-Biobank cohort used for the development of the eBMD_GRS_. This is most likely the reason why the eBMD_GRS_ in general was a stronger predictor of fracture risk and bone microstructure parameters than the 2 DXA-based GRSs. However, importantly both for bone site-specific fracture prediction and for association with cortical versus trabecular bone microstructure parameters, there were clear differences in the patterns of the relative importance of the different GRSs. The eBMD_GRS_ but not FN-BMD_GRS_ independently predicted trabecular bone thickness as well as vertebral and forearm fractures. In contrast, both FN-BMD_GRS_ and eBMD_GRS_ independently predicted cortical bone mass parameters (cortical area and cortical thickness) and hip fractures (Fig 4). We propose that eBMD_GRS_ captures unique information on trabecular bone microstructure useful for prediction of forearm and vertebral fractures. The strong prediction of trabecular bone parameters by eBMD_GRS_ is most likely related to the exceptional high trabecular bone content in the calcaneus (90%) where ultrasound eBMD is measured (29). In contrast, both FN-BMD_GRS_ and eBMD_GRS_ capture unique information on cortical bone mass useful for prediction of hip fractures (Fig 4).

There are some clear advantages to using GRS for the prediction of different health outcomes. First, GRSs do not change over the lifetime, enabling both early identification of individuals at high risk to be considered for primary prevention strategies and for later risk assessment in patients considered for secondary prevention strategies. In addition, genome-wide array genotyping has a relatively low one-time cost and can be used to calculate GRSs not only for fracture risk prediction but also for prediction of several other complex diseases (48–50).

The main strength with the present study is the large number of x-ray–verified forearm, hip, and vertebral fractures, enabling separate fracture prediction by bone site. In addition, detailed HRpQCT analyses at the distal radius were available in a subset of MrOS Sweden, enabling us to evaluate the combined performances of the GRSs for trabecular and cortical bone microstructure parameters separately at the bone site for forearm fractures. Although we aimed to determine the predictive value of the GRSs for the separate prediction of fractures at different bone sites, as well as for trabecular and cortical bone microstructure, a limitation is that information on clinical risk factors and FN-BMD were not available in the 2 large fracture cohorts used in the present study. Further studies in large cohort with a high fracture prevalence and information on clinical risk factors and available FN-BMD are warranted to determine if the herein-identified combinations of GRSs predict bone site-specific fracture risk beyond clinical risk factors and FN-BMD. Nevertheless, current fracture models in clinical use are not designed to discriminate between risk for fractures at different bone sites (13, 14). It is a limitation with the present study that we only used stepwise regression and not more modern methods such as LASSO or Bayesian Model Averaging. Another limitation with the present study is that only individuals of white ethnic background were included, and because the genetic background varies in different populations, we cannot generalize our findings to other race/ethnic populations.

In conclusion, the eBMD_GRS_ is the only independent GRS for prediction of forearm and vertebral fractures while both FN-BMD_GRS_ and eBMD_GRS_ contribute independent information for prediction of hip fractures. We propose that eBMD_GRS_ captures unique information of trabecular bone microstructure useful for the prediction of forearm and vertebral fractures. The findings in the present study may facilitate personalized medicine to predict different fracture types as well as cortical and trabecular bone microstructure parameters separately. Personalized medicine has the potential to customize therapy with the best response and highest safety margin to ensure better patient care, by enabling each patient to receive earlier diagnoses, risk assessments, and optimal treatments.

## Abbreviations

AIC: Akaike information criterion
AUC: area under the receiver operating characteristic curve
BMD: bone mineral density
BUA: bone ultrasound attenuation
CT: computed tomography
DXA: dual-energy x-ray absorptiometry
eBMD: estimated bone mineral density of the calcaneus
eBMD_GRS_: weighted genetic risk score for estimated bone mineral density of the calcaneus
FDR: false discovery rate
FN-BMD: femoral neck bone mineral density
FN-BMD_GRS_: weighted genetic risk score for femoral neck bone mineral density
GWAS: genome-wide association study
HRpQCT: high resolution peripheral quantitative computed tomography
IBS: identical-by-state
LS-BMD: lumbar spine bone mineral density
MrOS: Osteoporotic Fractures in Men
NSHDS: Northern Sweden Health and Disease Study
SNP: single nucleotide polymorphism
SOS: speed of sound
UFO: Umeå Fracture and Osteoporosis
vBMD: volumetric bone mineral density

## Additional Information

***Disclosure Summary:*** The authors have nothing to disclose.

***Data Availability:*** Restrictions apply to the availability of data generated or analyzed during this study to preserve patient confidentiality or because they were used under license. The corresponding author will on request detail the restrictions and any conditions under which access to some data may be provided.
